# Moxibustion for Primary Dysmenorrhea at Different Interventional Times: A Systematic Review and Meta-Analysis

**DOI:** 10.1155/2016/6706901

**Published:** 2016-12-28

**Authors:** Chao-qin Gou, Jing Gao, Chen-xi Wu, Ding-xi Bai, Hong-yuan Mou, Xiao-lin Hou, Xia Zhao

**Affiliations:** Chengdu University of Traditional Chinese Medicine, No. 37 Shierqiao Road, Jinniu District, Chengdu, Sichuan 610075, China

## Abstract

Primary dysmenorrhea (PD) is one of the most common diseases in gynecology at present. Some clinical trials have reported the effects of moxibustion and confirmed temporal factors are the important elements influencing the efficacy of moxibustion. However, no systematic review has yet been conducted. In this study, we assessed the effects of moxibustion in patients with PD enrolled in randomized controlled trials (RCTs) and the difference among different intervention times to start moxibustion. We extracted data for studies searched from 10 electronic databases and evaluated the methodological quality of the included studies. We discussed three outcomes: effective rate, pain remission, and the level of PGF2*α* in serum. Current clinical researches showed that, compared with nonmoxibustion treatments for PD, moxibustion leads to higher effective rate and lower level of PGF2*α* in serum. However, there was no difference in using moxibustion to treat PD at different intervention times. Based on the theory of Chinese medicine and the results of this study, choosing 5 ± 2 days before menstruation to start moxibustion can achieve good efficacy for PD patients. However, more high-quality RCTs are needed to confirm the conclusions.

## 1. Introduction

Primary dysmenorrhea (PD) is one of the most common diseases in gynecology at present [1]. It has a strong impact on women's lives, work, and study and represents a substantial public health burden [2–4]. Pain is the main symptom. It has two subcategories: primary and secondary dysmenorrhea. Studies reported the prevalence of PD was 20%–91% [5–7]; the women with severe pain accounted for 2%–29%. Because of unknown etiology, there is no definite cure for modern medicine at present; the main measure of PD is painkillers. The drugs most widely used are nonsteroidal anti-inflammatory drugs, which have been taken as the first-line therapy [8]. Undeniably, painkillers do relieve most women's pain in menstruation; however, they have a failure rate [9] of 20%–30%. What is more, drugs' subsequent effectiveness is poor and side effects are significant in some women. Therefore, more and more patients of PD are turning to complementary and alternative medicine, such as moxibustion.

Moxibustion is a kind of traditional Chinese therapy, selecting the acupoints to operate. Moxibustion includes moxibustion with moxa stick, moxa cone, needle warming moxibustion, and so on. TCM holds that PD is caused by cold accumulation, blood stasis, and qi stagnation; in a word, stagnation leads to pain. The moxibustion therapy based on the thermal effects, integrating radiation effects, and pharmacological actions of moxa to treat PD [10, 11], which was reported, has achieved good effects.

As one of the commonly used moxibustion therapies, moxibustion with moxa stick can be implemented by the methods of mild, bird-pecking, and circling moxibustion. Even though moxibustion has advantages, such as simple operation, no pain, and good subsequent effectiveness, it still needs evidence to convince us of some aspects [12–14]. In recent years, the experiments have confirmed that temporal factors are the important elements influencing the efficacy of moxibustion [15]; the interventional time is one of them. However, there are no definite evidences to determine whether the differences exist on clinical effects of moxibustion at different intervention times to treat and to provide the optimal interventional time for clinical patients with PD. So we conducted a systematic review to assess the efficacy of moxibustion for PD as well as to determine when is the optimal interventional time to start treatment, which can optimize the use of resources and therapeutic effect of moxibustion, and make further efforts to the standardization of moxibustion.

## 2. Methods

### 2.1. Data Sources

We conducted literature searches in the following databases from their respective dates of inception until March 2016: the Cochrane Central Register of Controlled Trials (CENTRAL), MEDLINE, EMBASE, The Allied and Complementary Medicine Database (AMED), Cumulative Index of Nursing & Allied Health Literature (CINAHL+), Web of Science, the Chinese Biological Medicine Database (CBM), China National Knowledge Infrastructure (CNKI), Chinese Scientific Journal Database (VIP), and WanFang Data. We also searched the ClinicalTrials.gov Database and Chinese Clinical Trial Register (ChiCTR) to collect potentially relevant clinical trials.

### 2.2. Inclusion Criteria

Studies will be included if they satisfy the following criteria: diagnosed with PD (according to the Primary Dysmenorrhea Consensus Guidelines [8]); all the parallel randomized controlled trials (RCTs) of moxibustion in which there were treatment group with pure moxa stick and control group with western medicine, traditional Chinese medicine, or acupuncture were included; the outcomes were pain assessment (evaluation tool is visual analogue scale (VAS)), effective rate (the effectiveness was evaluated referring to “criteria of diagnosis and therapeutic effect of diseases and syndromes in traditional Chinese medicine,” which is made by the State Administration of Traditional Chinese Medicine in China. The effectiveness includes three grades: ① cure: after treatment, lower abdominal pain and other accompanying symptoms disappeared and did not recur 3 menstrual cycles later after stopping treatment; ② effectiveness: lower abdominal pain and other accompanying symptoms relieved, such as nausea, vomiting, diarrhea, cold sweat, and peripheral coldness; ③ treatment failure: lower abdominal pain and other accompanying symptoms were not alleviated. Moxibustion is a kind of traditional Chinese therapy, so the effective rate that referred to this standard is appropriate, the effective rate = cases  (cure  +  effectiveness)/total  cases, and the level of PGF2*α* in serum. There were no restrictions on language, population characteristics, and publication type.

### 2.3. Data Extraction and Risk of Bias Assessment

Two reviewers independently perform the literatures searching (C. Q. Gou, X. Zhao), study selection (C. Q. Gou, X. L. Hou), and data extraction (C. Q. Gou, D. X. Bai). The data extraction form included general study characteristics, baseline characteristics of subjects, details of interventions management, outcomes, and adverse effect. The methodological quality for each included study was assessed according to the Cochrane Handbook [26] version 5.1.0 (C. Q. Gou, C. X. Wu). Publication bias was assessed quantitatively with Begg's test for the outcome of effective rate by using Stata 12.0 software. Disagreement was resolved by discussion and consensus.

### 2.4. Data Synthesis and Analysis

RevMan5.2 software provided by the Cochrane Collaboration was employed to perform the data analysis. Relative risks (RR) were calculated for the dichotomous outcome of effective rate; and mean differences (MD) were calculated for the continuous outcomes of pain assessment and the level of PGF2*α* in serum, both with 95% confidence intervals. Subgroup analysis was conducted for different interventional times. Heterogeneity was recognized as significant when *I*^2^≧50%. A Fixed-effect model was performed when there was no significant heterogeneity of the data; A random-effect model was used if significant heterogeneity existed. Publication bias was assessed quantitatively for the outcomes. Sensitivity analysis was performed by changing analysis model and statistics to test the stability on the results of data analysis.

## 3. Results

### 3.1. Study Description

**Figure 1:**
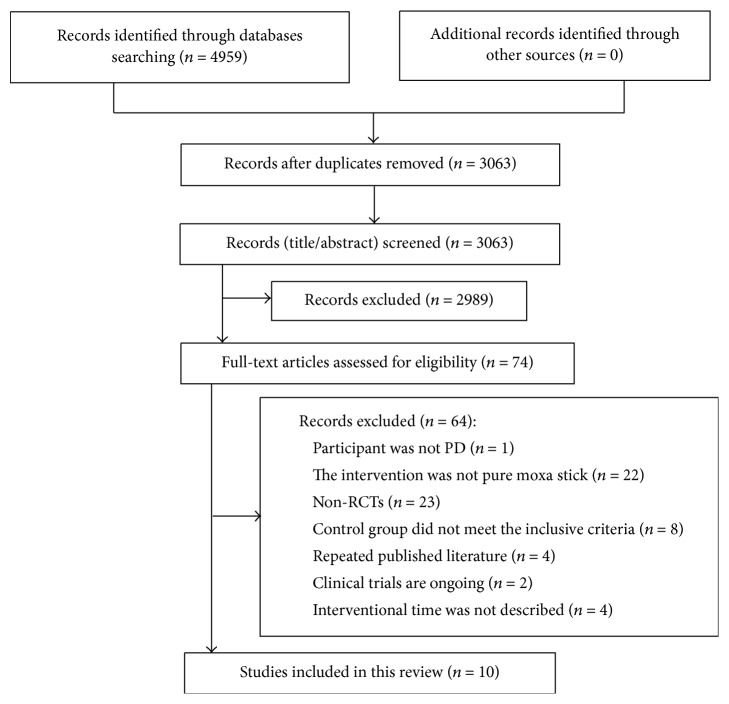
Flowchart of the trials selection process.

The process of study selection is summarized in Figure 1. After primary searches from the 10 databases, a total of 4959 records were screened. Records after duplication removed were 3063. By reading the titles and abstracts, 2989 records were excluded. Full-texts of 74 records were retrieved, and 64 articles were excluded with reasons listed as follows: participant was not PD (*n* = 1), the intervention was not pure moxa stick (*n* = 22), research was not RCT (*n* = 23), control group did not meet the inclusive criteria (*n* = 8) and duplicate publication (*n* = 4), clinical trials are ongoing (*n* = 2), and interventional time was not described (*n* = 4). In summary, 10 studies were eligible and included in the meta-analysis finally.

### 3.2. General Study Characteristics

The general characteristics of included trials are presented in Table 1. All the RCTs included were published in Chinese, 625 patients with PD in total and 19 patients dropped out. The included patients' age ranges from 13 to 36 years old. The degree of disease was mainly moderate to severe. The duration of PD ranged from half a year to 15 years. The main symptoms were cold and damp accumulation,* qi* stagnation, and blood stasis. All the RCTs reported the therapeutic interventional time, in which 2 trials [17, 18] chose 3 days before menstruation to start the treatment, 3 trials [19–21] chose 5 days before, 4 trials [22–25] chose 7 days before, and 1 study [16] reported 2 weeks before menstruation to start the treatment. The total course of all the trails included was 3 menstrual cycles.

### 3.3. Risk of Bias Assessment

Results of the risk of bias assessment are presented in Table 2. Six trials [16, 17, 22–25] reported the sequence generation for randomization using the table of random; only 2 trials [17, 23] mentioned the allocation concealment with envelope. Limited by insufficient information, we could not judge whether it was conducted properly or not. Because of the particularity of moxibustion with moxa stick comparing to medicine, patients and healthcare providers could not be blinded. The blinding of data collectors, outcome assessment, and data analysts was reported in 2 studies [22, 23] and in the remaining 8 studies was unclear. One trial [23] mentioned dropouts. All studies were free of selective reporting. Adverse events were poorly reported in all studies but two [22, 23]. Publication bias was assessed quantitatively for the outcome of effective rate. The other studies were judged as unclear because of the limited number of trials.

## 4. Outcomes

### 4.1. Effective Rate

**Figure 2:**
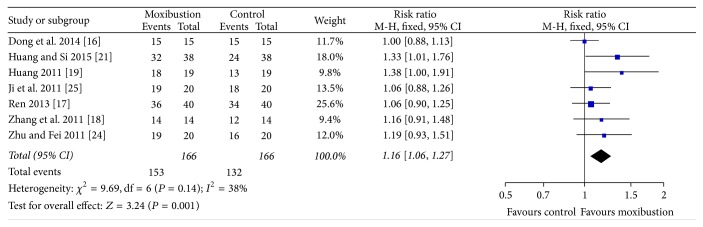
Analyses of the total effective rate.

The effective rate of clinical treatment was pooled for 7 studies. Heterogeneity among studies was low (*P* = 0.14, *I*^2^ = 38%); thus, a fixed-effect model was employed. There was significant difference between moxibustion and non-moxibustion treatments on increasing the total effective rate (RR = 1.16, 95% CI (1.06, 1.27), *P* = 0.001) (Figure 2).

### 4.2. Pain Remission and PGF2*α* Levels

**Figure 3:**
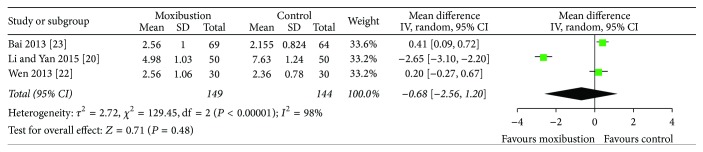
Pain remission after moxibustion versus nonmoxibustion treatments (control) for PD.

Three studies [20, 22, 23] used visual analogue scale (VAS) to evaluate treatment effects on pain. Heterogeneity among studies was high (*I*^2^ = 98%), but it could be explained, so random effect model was used for statistical analysis. The studies showed no statistical significance to reduce the pain of patients with PD between moxibustion and nonmoxibustion treatments (MD = −0.68, 95% CI (−2.56, 1.20), *P* = 0.48) (Figure 3).

**Figure 4:**
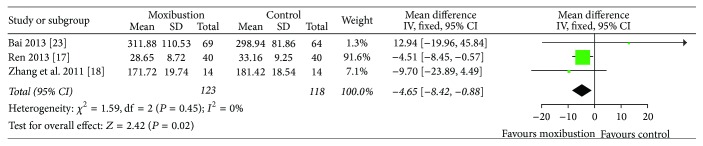
Change of PGF2*α* levels in serum after moxibustion versus nonmoxibustion treatments (control) for PD.

When it comes to PGF2*α* levels, 3 studies [17, 18, 23] showed no heterogeneity in the results (*I*^2^ = 0%). Therefore, a fixed-effect model was employed. The meta-analysis showed that there was significant difference on the levels of PGF2*α* in serum (MD = −4.65, 95% CI (−8.42, −0.88), *P* = 0.02) (Figure 4) between moxibustion and nonmoxibustion treatments.

**Figure 5:**
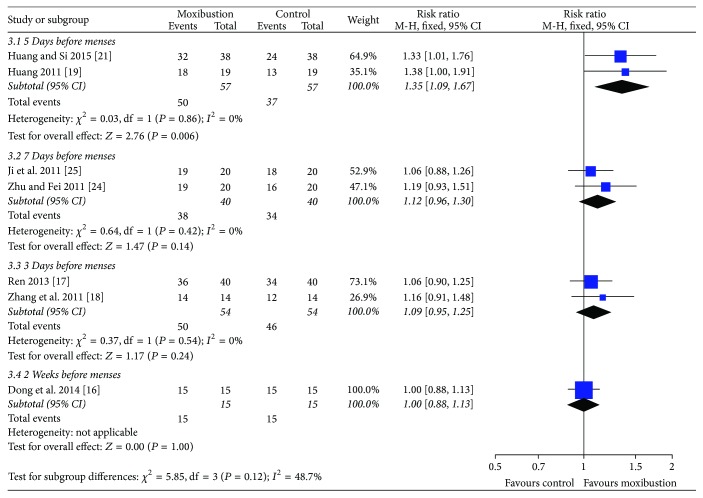
Change in effective rate with moxibustion at different interventional times for PD.

*Subgroup Analysis*. In order to analyze if there was difference on clinical curative effect of moxa moxibustion at different intervention times to treat PD, subgroup analysis was performed for the outcome of effective rate. The subgroup meta-analysis showed there was no statistical significance among 3 days, 5 days, 7 days, or two weeks before menstruation to start the moxa moxibustion therapy on improving the effective rate (*P* = 0.12) (Figure 5).

### 4.3. Sensitivity Analysis

Sensitivity analysis was performed by changing analysis model and statistics. The studies showed the results of the meta-analysis conducted before being stable.

### 4.4. Publication Bias

Seven studies' publication bias was assessed for the outcome of effective rate by using Stata 12.0 software. Begg's test results showed there was no publication bias [*z* = 1.20 (continuity corrected) Pr > |*z*| = 0.230 (continuity corrected), 0.230 > 0.05]. The other studies' publication bias was judged as unclear because of the limited number of trials.

### 4.5. Adverse Events

Two studies reported the adverse effect. Six patients in total were found scald and halo moxibustion; no other specific symptoms and signs were checked out. The remaining 8 trials did not mention it at all.

## 5. Discussion

This systematic review showed that, comparing with nonmoxibustion treatments, moxibustion was more effective to increase the total effective rate and reduce the level of PGF2*α* in serum; it leads to higher total effective rate and lower level of PGF2*α* in serum but showed no statistical significance to reduce the pain of patients with PD. Listijo's study [27], in which he compared pure moxibustion with Chinese patent medicine or western medicine, has drawn the same conclusion that moxibustion did own some advantages to a certain extent. Researches of Jian-bin Zhang et al. [28, 29] indicated that the clinical effects of moxibustion contained two aspects: warming dredging and warming reinforcing; they have mutually influence. Therefore, moxibustion has its unique advantages in dredging and activating the meridian, nourishing Yin and benefiting blood. In traditional Chinese medicine, the main cause of PD is that “stagnation leads to pain” or “loss of nourishment leads to pain.” Based on warm stimulation, moxibustion adopted dredging the merdians, tonifying qi, and nourishing blood to treat PD against its pathogenesis and achieved the goal dispelling cold by warming the meridian, harmonizing Qi and blood, and promoting blood circulation to remove blood stasis. Thus it has acquired good clinical efficacy and improved the total effective rate of PD patients. PGF2*α* is a kind of substance that can cause pain. Nonsteroid anti-inflammatory drugs can relieve the pain of PD patients by inhibiting the synthetize and release of PGF2*α*. This review showed that moxibustion can lead to lower level of PGF2*α* in serum, which indicated that the efficacy of moxibustion may be related to the reduced level of PGF2*α* in serum. However, this study showed no significant difference to reduce the pain of patients with PD between moxibustion and nonmoxibustion treatments. This conclusion corresponded to Bai [23] and Wen's studies [22], which showed that moxibustion's immediate effect of pain relieving was not as obvious as drugs, but as time went by, the long-term efficacy of moxibustion was better.

The interventional time is one of the most important factors that can influence the efficacy of moxibustion.* Huang di's Canon of Medicine* says that to cure disease at its optimal time will achieve the best efficacy, different interventional times lead to different efficacy. As chronomedicine rises, the interventional time is becoming more and more important. Therefore, this study performed a subgroup analysis for the interventional time in patients with PD to determine whether there was difference on clinical effects of moxibustion at different intervention times to start treatment. The result showed that there was no statistical significance among 3 days, 5 days, 7 days, or two weeks before menstruation to start the moxibustion therapy on improving the effective rate. The fact that the total amount of moxibustion cannot lead to the qualitative change may be the reason. Mei Zhang et al. [30] conducted a research to observe the effect of different interventional times of moxibustion on the intensity of uterine contraction in dysmenorrhea rats. They found that the interventional time did influence the efficacy of moxibustion in treating PD. However, there was no difference in biological indicators between earlier intervention and immediate intervention when patient was in pain that corresponded to our study.

At present, moxibustion therapy for PD is inclined to start treating before menstruation. The reasons are as follows: in modern medicine, the main pathogenesis of PD is the increase of prostaglandin (PG) and other hormones [31, 32]. 48 hours before menstruation is the peak period of producing PG in endometria. Treating PD before menstruation is in accord with its pathological changes. Traditional Chinese medicine advocates “preventive treatment of disease.” Moxibustion adopts the warm stimulation to inspire the* Qi* of meridian through the acupoints, in order to strengthen healthy* Qi* to resist all kinds of pathologic* Qi* when in health or before the occurrence of disease and achieve the purpose of health care and disease prevention, and to reduce the subsequent diseases' damage. This is the essence of “preexcitation stress theory”: to give the body an optimal stimulation in advance, so as to produce adaptive stimulus and start the endogenous protective mechanism to produce a positive regulatory effect. To treat PD before menstruation is in line with “preventive treatment of disease” in traditional Chinese medicine. The interventional time can be determined according to regular pattern of human physiological and pathological changes and timing effect theory, and so on.

In conclusion, female menstruations are affected by many factors, so the exact date of their menstruation is unable to be predicted accurately. Based on the theory above and the results of subgroup analysis, we found that choosing 5 ± 2 days before menstruation to start treatments can achieve good efficacy for PD patients.

### 5.1. Strengths and Limitations of Study

To our knowledge this is the first meta-analysis of randomized controlled trials directly comparing moxibustion with nonmoxibustion treatments for PD and analyzing the differences to start moxibustion at different interventional times. We discussed the efficacy of moxibustion synthetically by adopting three kinds of outcomes: effective rate, pain remission, and the level of PGF2*α*. As we all know, the main symptom of dysmenorrhea is pain, which can be accompanied by other symptoms. In my systematic review, the degree of PD included by study was mainly moderate to severe; that is to say, they are more likely to exist accompanying symptoms; only the outcome of pain is not enough, while the outcome of effective rate evaluated not only the pain, but also the accompanying symptoms. Therefore, I chose the “effective rate” as supplement.

Some limits of this study should be considered: firstly, the included studies were all in Chinese (there are 2 clinical trials, the protocol of which in English met the inclusion criteria, but the topic has not yet been completed and paper has not yet been published up to the time limit), so language bias may exist. Secondly, the number of studies in subgroup analysis is small; therefore, the application of the research conclusion will be limited. Thirdly, the overall risk of bias was evaluated as unclear, which may lead to low reliability of the research results at some extent.

## 6. Conclusions

In conclusion, current clinical research shows that, compared with nonmoxibustion treatments for PD moxibustion leads to higher total effective rate and lower level of PGF2*α* in serum. However, there was no difference in using moxibustion to treat PD at different intervention times. Based on the theory above and the results of subgroup analysis, we found that choosing 5 ± 2 days before menstruation to start treatments can achieve good efficacy for PD patients. Because the overall risk of bias was evaluated as unclear, application of the conclusions needs to be cautious. With the specificity of the operation technology of traditional Chinese medicine, patients and healthcare providers could not be blinded. Hence, separating the researchers, operators and outcome measurers as well as expanding the sample size of trials can improve the methodological quality of researches on Traditional Chinese Medicine.

## Figures and Tables

**Table 1:** General characteristics of included trials.

Study	Sample (T/C)	Age	Duration (year)	Symptom	Interventional time (premenstrual)	Control	Course of treatment (month)	Outcomes
Dong et al. 2014 [16]	15/15	18~23	NR	Q	14 days	Acupuncture	3	①
Ren 2013 [17]	40/40	16~28	1~8	H	3 days	Medicine	3	① ③
Zhang et al. 2011 [18]	14/14	15~24	0.5~6	NR	3 days	Medicine	3	① ③
Huang 2011 [19]	19/19	13~18	0.4~3	NR	5 days	Medicine	3	①
Li and Yan 2015 [20]	50/50	16~27	2~9	NR	5 days	Medicine	NR	②
Huang and Si 2015 [21]	38/38	15~27	0.5~5	NR	5 days	Medicine	3	①
Wen 2013 [22]	30/30	15~36	1~20	QH	7 days	Medicine	3	②
Bai 2013 [23]	69/64	13~35	NR	QH	7 days	Medicine	3	② ③
Zhu and Fei 2011 [24]	20/20	17~28	0.2~10	H	7 days	Medicine	3	①
Ji et al. 2011 [25]	20/20	18~24	1~10	NR	7 days	Acupuncture	3	①

NR = not reported; Q = *qi* stagnation and blood stasis; H = cold and damp accumulation; ① = effective rate; ② = pain assessment; ③ = PGF2*α* levels.

**Table 2:** Summary of risk of bias assessment for studies included.

Study	Random sequence generation	Allocation concealment	Blinding of participants and personnel	Blinding of outcome assessment or statistician	Complete outcome data	Selective reporting	Other sources of bias
Dong et al. 2014 [16]	**+ **	**?**	**?**	**?**	**+**	**+**	**?**
Ren 2013 [17]	**+**	**+**	**?**	**?**	**+**	**+**	**?**
Zhang et al. 2011 [18]	**?**	**?**	**?**	**?**	**+**	**+**	**?**
Huang 2011 [19]	**?**	**?**	**?**	**?**	**+**	**+**	**?**
Li and Yan 2015 [20]	**?**	**?**	**?**	**?**	**+**	**+**	**?**
Huang and Si 2015 [21]	**?**	**?**	**?**	**?**	**+**	**+**	**?**
Wen 2013 [22]	**+**	**?**	**?**	**+**	**+**	**+**	**?**
Bai 2013 [23]	**+**	**+**	**+**	**+**	**+**	**+**	**?**
Zhu and Fei 2011 [24]	**+**	**?**	**?**	**?**	**+**	**+**	**?**
Ji et al. 2011 [25]	**+**	**?**	**?**	**?**	**+**	**+**	**?**

“+” **= **low risk of bias; “?” **= **unclear risk of bias.
